# Risk of Hemorrhage during Needle-Based Ophthalmic Regional Anesthesia in Patients Taking Antithrombotics: A Systematic Review

**DOI:** 10.1371/journal.pone.0147227

**Published:** 2016-01-22

**Authors:** Augusto Takaschima, Patricia Marchioro, Thiago M. Sakae, André L. Porporatti, Luis André Mezzomo, Graziela De Luca Canto

**Affiliations:** 1 Florianópolis Hospital, Florianópolis, Brazil; 2 Sianest—Anesthesiology Service, Florianópolis, Brazil; 3 Brazilian Centre for Evidence-based Research, Health Sciences Centre, Federal University of Santa Catarina, Florianópolis, Brazil; 4 Unisul—Universidade do Sul de Santa Catarina, Tubarão, Brazil; 5 University of São Paulo, Bauru, Brazil; 6 Faculty of Medicine and Dentistry, University of Alberta, Edmonton, Alberta, Canada; Massachusetts Eye & Ear Infirmary, Harvard Medical School, UNITED STATES

## Abstract

**Background:**

Patients undergoing ophthalmic surgery are usually elderly and, due to systemic disease, may be on long-term therapy, such as antithrombotic agents. Rates of hemorrhagic complications associated with invasive procedures may be increased by the use of anticoagulants and antiplatelet agents.

**Objective:**

To compare the incidence of hemorrhagic complications in patients undergoing needle-based ophthalmic regional anesthesia between patients on antithrombotic therapy and those not on such therapy.

**Methods:**

A systematic review was conducted by two independent reviewers based on searches of Cochrane, LILACS, PubMed, Scopus, Web of Science, and the “gray” literature (Google Scholar). The end search date was May 8, 2015, across all databases.

**Results:**

Five studies met the eligibility criteria. In three studies, individual risk of bias was low, and in two of them, moderate. In all studies, no differences regarding mild to moderate incidence of hemorrhagic complications were found between patients using antithrombotics (aspirin, clopidogrel, and warfarin) and those not using them. Rates of severe hemorrhagic complication were very low (0.04%) in both groups, supporting the safety of needle blocks, even in patients using antithrombotics. High heterogeneity across studies prevented meta-analysis. Limitations to these results include low statistical power in three experimental studies and a large 95% confidence interval in the two retrospective cohorts.

**Conclusion:**

In this review, none of the selected studies showed significant bleeding related to needle-based ophthalmic regional anesthesia in association with the use of aspirin, clopidogrel, or vitamin K inhibitors. Since the available data is not powerful enough to provide a reliable evaluation of the true effect of antithrombotics in this setting, new studies to address these limitations are necessary.

## Introduction

Patients undergoing ophthalmic surgery are usually elderly and often undergo long-term therapy for systemic diseases, including cardiac problems [1–4]. Prevention of adverse cardiovascular events often involves the chronic usage of antithrombotic drugs, such as anticoagulants and antiplatelet agents. In a study evaluating 48,862 patients undergoing cataract surgery, 28.1% were on aspirin, 5.1% on warfarin, and 1.9% on clopidogrel [2].

Rates of hemorrhagic complications associated with invasive procedures may be increased by the use of anticoagulants and antiplatelet agents [3]. Some studies [2, 5] have reported a higher incidence of subconjunctival hemorrhage in patients using antithrombotics when undergoing ophthalmic regional anesthesia. Reduction of this risk of hemorrhage can sometimes be achieved with topical anesthesia [6], although for some ocular procedures, this technique might not be an option [7]. Considering that it is now rare for general anesthesia to be used during ophthalmic surgery [1], many patients on antithrombotics who have ophthalmic surgeries undergo needle-based regional anesthesia [7].

Discontinuation of anticoagulants and antiplatelet agents may also reduce the incidence of perioperative hemorrhagic events, in some cases at the expense of increased thrombotic risk [3]. Regarding antiplatelet therapy, a systematic review reported that up to 10.2% of acute cardiovascular adverse events were preceded by aspirin withdrawal [8], likely because withdrawal results in a rebound effect as platelet aggregation increases [9]. In addition, standard guidelines for percutaneous coronary intervention stress the importance of 12 months of dual antiplatelet therapy after placement of a drug-eluting stent and warn of the hazards of premature discontinuation [10, 11].

In other settings, this increase in the number of thrombotic events is not as clear. Discontinuation of oral anticoagulation in patients with atrial fibrillation does not seem to raise thrombotic risk compared to those undergoing perioperative bridging with low-molecular-weight heparin after stopping oral anticoagulants [12]. Although for other indications, such as prosthetic heart valves, bridging of heparin or even maintenance with anticoagulation is still advisable [3].

Previous reviews evaluating hemorrhagic complications with respect to ophthalmic surgery have suggested that it is safe to continue antithrombotic therapy before facetectomy and vitrectomy [1, 7]. Assessing patients undergoing 25-gauge vitrectomy, Mason et al. [13] found no difference in rates of surgical hemorrhagic complications between those using clopidogrel or warfarin and those not using either. Kobayashi et al. [14] assessed aspirin and warfarin as a risk factor for hemorrhage during phacoemulsification^;^ no significant difference in the incidence of intraoperative or postoperative hemorrhagic complications was reported between users and non-users of these agents, supporting the maintenance of aspirin and warfarin before cataract surgery.

Risk of bleeding related to ophthalmic anesthesia may be increased in patients receiving antithrombotic therapy [2]. To our knowledge, no systematic review has yet summarized the available evidence on this subject. Thus, the objective of this review was to determine whether rates and types of hemorrhagic complications related to needle-based ophthalmic block differ between patients using antithrombotics and those not using these agents.

## Methods

This systematic review was written in accordance with the Preferred Reporting Items for Systematic Reviews and Meta-Analyses (PRISMA) checklist [15].

### Protocol and registration

The systematic review protocol was registered at the International Prospective Register of Systematic Reviews (PROSPERO) under number CRD42015023182.

### Terminology

For this systematic review, needle-based ophthalmic block was defined as peribulbar or retrobulbar anesthesia. Sub-Tenon anesthesia was not considered needle block. Antithrombotic agents included antiplatelet and anticoagulant agents.

Whenever possible, hemorrhagic complications related to anesthesia were assessed on the 4-grade scale of hemorrhage developed by Kallio et al. [16] (grade 1 = spot ecchymosis; 2 = lid ecchymosis involving half of the lid surface area or less; 3 = lid ecchymosis all around the eye with no increase of the intraocular pressure; 4 = retrobulbar hemorrhage with increased intraocular pressure). Hemorrhages of grade 1 or 2 were considered mild; grade 3, moderate; and grade 4, severe.

### Study design

#### Information sources

Studies to be considered for inclusion were identified by searching the following electronic bibliographic databases: Cochrane, LILACS, PubMed, Scopus, and Web of Science. An additional search of the “gray literature” was performed using Google Scholar. The end search date was May 8, 2015, across all databases. In addition, the reference lists of the selected articles were searched manually.

Appropriate truncation and word combinations were selected and adapted for each database search (S1 Table), with the aid of a health sciences librarian. All references were run through the reference manager software EndNote^®^ (Thomson Reuters, Philadelphia, PA, USA), and duplicate hits were removed.

#### Study selection and eligibility criteria

We reviewed studies whose objective was to compare the incidence of hemorrhagic complications associated with peribulbar or retrobulbar anesthesia between patients using antiplatelet agents and anticoagulants and patients not using such agents.

There were two phases of review. In phase 1, we reviewed titles and abstracts and excluded the following: (1) studies conducted in infants (0–18 years) and (2) reviews, letters, conference abstracts, and editorials. In phase 2, we reviewed full-text articles and additionally excluded the following: (3) studies evaluating only the sub-Tenon’s block or not differentiating it from other ophthalmic regional anesthesia techniques, (4) studies assessing surgery-related hemorrhagic complications and not anesthesia-related ones, and (5) studies where it was not clearly stated if patients used antithrombotic agents or without a group control.

Two authors (A.T., P.M.) independently reviewed all search results. In both phases, when disagreements emerged between the two reviewers, they tried to reach a consensus. When they were unable to reach an agreement, a third author (T.M.S.) made the final decision. Articles that did not appear to meet the inclusion criteria were discarded. In phase 2, the same authors reviewed the full-text of the articles. The third author (T.M.S.) read the abstracts of all the selected articles and made the final decision on inclusion; however, final selection was always based on the full text of the publication. The reference lists of selected studies were critically assessed by both A.T. and P.M.

#### Data collection

One author (A.T.) collected data from the selected studies. The following information was recorded: study background (authors, year, country, study design), population characteristics (number of patients, mean age), intervention characteristics (presence of a control group, presence of patients on antithrombotics), outcome characteristics (incidence, confidence interval of incidence, and severity of bleeding), and main conclusions. A second author (P.M.) crosschecked all the collected information and confirmed its accuracy. Again, any disagreement was resolved by discussion and mutual agreement among the three reviewers (A.T., P.M., T.M.S.).

#### Risk of bias in individual studies

The methodology of the selected studies was evaluated using the Meta-analysis of Statistics Assessment and Review Instrument (MAStARI) from the Joanna Briggs Institute [17]. Two reviewers (A.T., P.M.) independently assessed the quality of each included study. Disagreements between the reviewers were resolved through discussion.

#### Outcome measures

Incidence of hemorrhagic complications related to needle-based ophthalmic anesthesia was considered the main outcome. However, other types of outcomes measured were also noted.

#### Synthesis of results and risk of bias across studies

If possible, the possibility of meta-analysis and risk of bias across studies was considered.

#### Additional analyses

A post-hoc analysis was conducted with G*Power 3.1.9.2 (Faul, Erdfelder, Lang, & Buchner, 2007) for each individual experimental study to evaluate the power to exclude type II errors.

## Results

### Study selection

**Fig 1:**
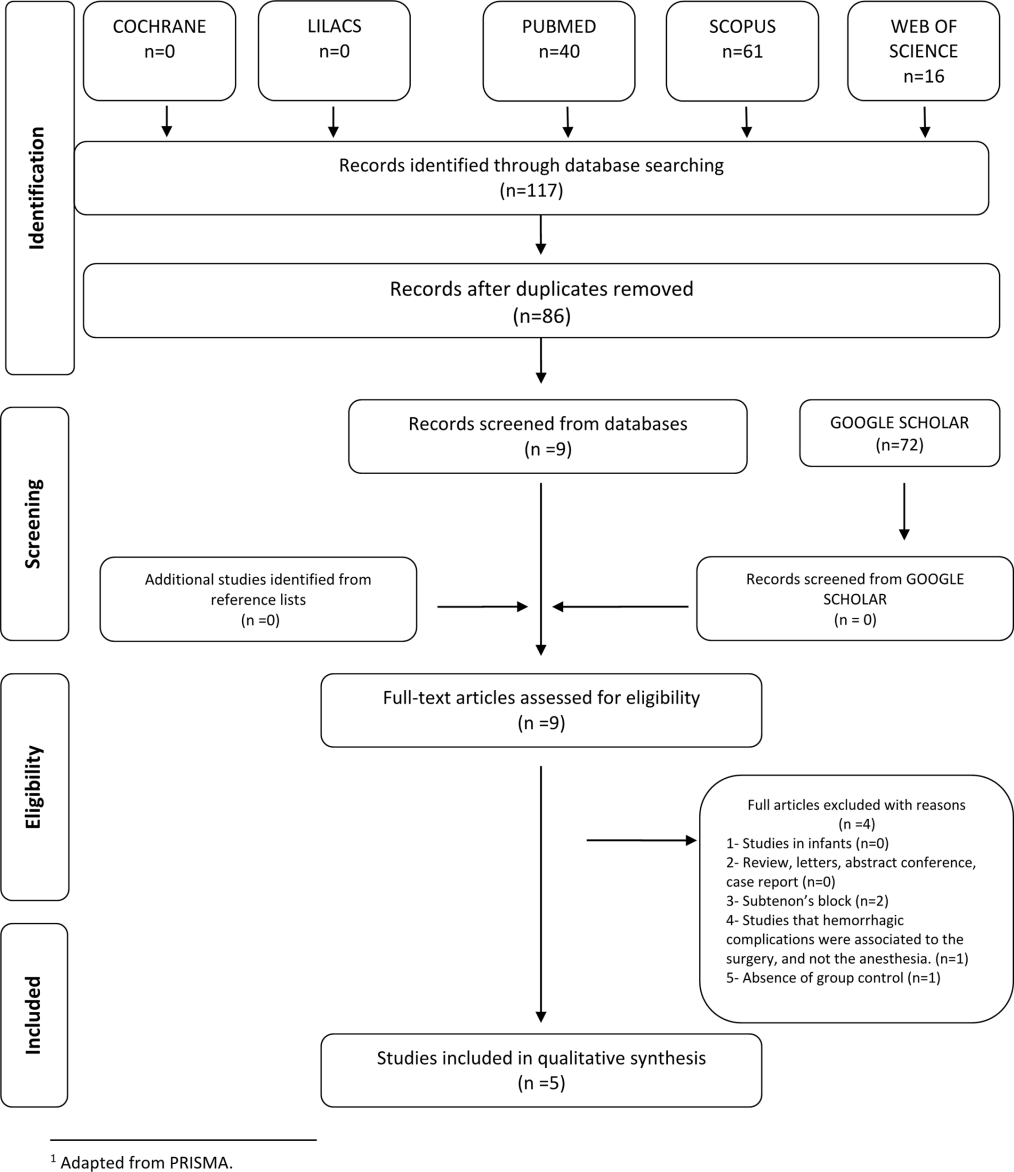
Flow diagram of literature search and selection criteria.

During the initial search (phase 1) and following duplicate removal, 86 different citations were identified across the 5 electronic databases. An additional search using Google Scholar found no additional relevant articles. After a comprehensive evaluation of the abstracts, 9 articles were deemed potentially relevant and were selected for phase 2 assessment. Of these remaining studies, 4 were subsequently excluded (S2 Table). Thus, only 5 studies were retained for the final selection. A flowchart of the process of study identification and selection is shown in Fig 1.

### Study backgrounds

The 5 studies (2 cohort studies, 3 experimental) that addressed the review objective had an effective sample size of 24,332 patients (7,762 in intervention groups and 16,570 in control groups), most of them elderly. Three studies examined the effects of acetylsalicylic acid (ASA), 3 involved vitamin K antagonists, and 1 involved clopidogrel. A summary of the study backgrounds can be found in Table 1.

**Table 1:** Summary of included studies.

STUDY BACKGROUND			POPULATION CHARACTERISTICS		INTERVENTION CHARACTERISTICS					OUTCOME CHARACTERISTICS		
Author, year, country	Study design	Objective	Total *n*	Age mean/range (years)	Intervention (n)	Control (n)	Follow-up period	Primary outcomes	Statistical analysis	Incidence of bleeding	Severity of bleeding	Main conclusion
Calenda et al., 2011 [18], France	NRCT	To assess the safety of peribulbar block in patients treated with ASA [aspirin]	1,000 patients	Group A: 72, Group B: 70	Group A: patients on chronic aspirin therapy [not stopped] scheduled for eye procedures [n = 500]	Group B: patients who had never been treated with aspirin scheduled for an eye procedure [n = 500]	Bleeding was assessed until the 24th hour after peribulbar anesthesia	Hemorrhage related to peribulbar anesthesia, evaluated according to Kallio’s gradation*	Fisher’s exact test	Group A: 6%, Group B: 4.20%	Group A: Grade 1 = 6%; Group B: Grade 1 = 4%, Grade =: 0.20%, No grade 3 or 4 hemorrhage was encountered	Between the groups with and without preoperative ASA, the rate of eyelid hemorrhage in peribulbar block was not significantly different.
Calenda et al., 2012 [19], France	NRCT	To assess the safety of peribulbar block in patients treated with clopidogrel	2,000 patients	Group A: 71, Group B: 71	Group A: patients on chronic clopidogrel therapy (not stopped) scheduled for eye procedures (n = 1,000)	Group B: patients who had never been treated with clopidogrel scheduled for eye procedures (n = 1,000)	Bleeding was assessed until the 24th hour after peribulbar anesthesia	Hemorrhage related to peribulbar anesthesia, evaluated according to Kallio’s gradation*	Fisher’s exact test	Group A: 3%, Group B: 2%	Group A: Grade 1 = 3%; Group B: Grade 1 = 2%, No grade 3 or 4 hemorrhage was encountered.	Clopidogrel was not associated with a significant increase in potentially sight-threatening local anesthetic complications.
Calenda et al., 2014 [20], France	NRCT	To assess the safety of peribulbar block in patients treated with oral anticoagulants (VKA)	1,500 patients	Group A: 76, Group B: 71	Group A: patients on chronic oral anticoagulant (VKA) therapy (not stopped) scheduled for eye procedures (n = 750)	Group B: patients who had never been treated with oral anti-coagulants scheduled for eye procedures (n = 750)	Bleeding was assessed until the 24th hour after peribulbar anesthesia	Hemorrhage related to peribulbar anesthesia, evaluated according to Kallio’s gradation*	Fisher’s exact test	Group A: 2%, Group B: 1.75%	Group A: Grade 1 = 1.74%, Grade 2 = 0.26%; Group B:Grade 1 = 1.60%, No grade 3 or 4 hemorrhage was encountered	Oral anti-coagulants were not associated with a significant increase in potentially sight-threatening local anesthetic complications.
Kallio et al., 2000 [16], Finland	Prospective cohort	To assess the safety of the peribulbar or retrobulbar block in patients treated with warfarin (VKA) non-steroidal anti-inflammatory drugs and oral steroids	1,368 patients	Not reported	Group A1: warfarin [76], Group A2: aspirin stopped 0–2 days (139), Group A3: aspirin stopped 3–14 days [343]	Group B: patients not on anti-thrombotics (n = 609)	Bleeding was assessed until the 10th minute after ophthalmic block	Hemorrhage related to peribulbar anesthesia, evaluated according to Kallio’s gradation*	Fisher’s exact test	Group A1: 3.90%, Group A2: 5%, Group A3: 3.2%, Group B: 4.10%	For all groups—Grade 1: 2.4%, Grade 2 to 3: 1.6%, No grade 4 complication was encountered	The preoperative use of ASA or warfarin, whether or not it was discontinued, did not predispose to hemorrhage associated with peribulbar or retrobulbar block.
Katz et al., 2003 [21], USA/Canada	Prospective cohort	To estimate the risks and benefits associated with continuation of anticoagulants or antiplatelet medications before cataract surgery	19,283 cataract surgeries	Not reported	Group A1: cataract surgeries in which patients were routine aspirin users, discontinued, n = 977 (774]‡; Group A2: cataract surgeries in which patients were routine aspirin users, continued, n = 3363 [2407]‡; Group A3: cataract surgeries in which patients were routine warfarin users, discontinued. n = 208 [174]‡; Group A4: cataract surgeries in which patients were routine warfarin users, continued, n = 526 [348]‡	Group B1: cataract surgeries in which patients were not routine aspirin users, n = 14,322 [10,555]‡; Group B2: cataract surgeries in which patients were not routine warfarin users, n = 18,215 [13,434]‡	The anesthesiologist or nurse anesthetist recorded any intra-operative medical events. Patients were telephoned 7 days after surgery. There were 7,231 surgeries in the four clinical centers for which the results of a 24-hour ocular examination were recorded.	Ocular hemorrhage—hyphema, vitreous hemorrhage and retrobulbar hemorrhage [among injection anesthesia surgeries]	Fisher’s exact test	Group A1: 0.00%, Group A2: 0.04%, Group A3: 0.00%, Group A4: 0.00%, Group B1: 0.04%, Group B2: 0.04%	For all groups, only retrobulbar hemorrhages were considered as a complication of anesthesia; other hemorrhagic events are not related to needle-based ophthalmic block.	The risks of medical and ophthalmic events surrounding cataract surgery were so low that absolute differences in risk associated with changes in routine anticoagulant or antiplatelet use were minimal.

ASA, acetylsalicylic acid; NRCT, nonrandomized controlled trial; VKA, vitamin K antagonist

*Severity of bleeding according to the terminology described in the Methods section of the manuscript: 1, spot ecchymosis; 2, eyelid ecchymosis involving half of the lid surface area or less; 3, eyelid ecchymosis all around the eye with no increase in intra-ocular pressure; 4, retrobulbar hemorrhage with increased intraocular pressure

^‡^n = topical anesthesia + peribulbar or retrobulbar anesthesia (from total anesthesia, only peribulbar or retrobulbar)

### Risk of bias within studies

According to the MAStARI risk of bias assessment tool, three of the chosen studies had low risk of bias, 2 had moderate risk, and none had high risk. However, none of the studies fulfilled all methodological quality criteria. Studies with low risk of bias allow a better evaluation of the intervention effect. On the other hand, studies with moderate or high risk of bias could overestimate or underestimate the true effect of intervention [17]. More information can be found in Table 2 (concise assessment) and S3 Table (detailed assessment).

**Table 2:** Risk of bias: concise assessment.

**Author**	**Risk of Bias**
Calenda et al. [18]	Low
Calenda et al. [19]	Low
Calenda et al. [20]	Low
Kallio et al. [16]	Moderate
Katz et al. [21]	Moderate

### Results of individual studies

The results of the 5 studies evaluating hemorrhagic complications related to needle-based ophthalmic block in patients using antithrombotics are reported in detail in Table 1. In a retrospective cohort study, Kallio et al. [16] evaluated hemorrhagic complications associated with retrobulbar or peribulbar anesthesia in 1,383 patients using or not using antithrombotics who were scheduled for ophthalmic surgeries. Incidence of mild to moderate hemorrhagic complications (grades 1 to 3) showed no significant differences among patients who used antithrombotics and those who did not [warfarin, 3.9% (95% CI 0.8–11.1); ASA, 3.7% (95% CI 2.2–5.8); no antithrombotics, 4.1% (95%CI: 2.52–5.74)]. Severe hemorrhagic complications (grade 4) did not occur in any group.

In three different experimental studies, Calenda et al. [18–20] also found mild to moderate hemorrhagic complications (Kallio grades 1 to 3). The incidence of such complications occurring in association with ophthalmic anesthesia was compared for ASA [18], clopidogrel [19], and vitamin K inhibitors [20] with a nonrandomized control (no antithrombotic agents). The incidence of mild to moderate hemorrhagic complications was 6% (95% CI 4.08–8.45) among patients using ASA [18], 3% (95% CI 2.03–4.25) for clopidogrel users [19], and 2% (95% CI 1.12–3.27) for vitamin K antagonist users [20]. The incidence did not significantly differ between treatment and control groups in any case [18–20]. For vitamin K antagonist users [20], an international normalized ratio (INR) of prothrombin time evaluating the anticoagulation status, ranging from 1 to 4.5 (INR subgroups: 1–2, 2–3, 3–4, and 4–4.5), did not increase the incidence of hemorrhagic complications [20]; patients with an INR above 4.5 were excluded from the study due to the high hemorrhagic risk for both surgery and regional anesthesia. Severe hemorrhagic complications (grade 4) were not found in any of these studies.

A retrospective cohort study by Katz et al. [21] that evaluated 19,283 cataract surgeries was the only study reporting severe hemorrhagic complications. The incidence of retrobulbar hemorrhage was estimated to be 0.04% (95% CI: 0.001–0.10) in 14,823 patients submitted to peribulbar or retrobulbar anesthesia. This incidence did not differ significantly between patients using and not using ASA.

### Risk of bias across studies and synthesis of results

Due to high heterogeneity (study design and outcome) among the studies that could be compared, the synthesis of results was considered unreliable.

### Additional analyses

From the included studies, three were considered experimental and underwent a post-hoc analysis power analysis. All of the studies had a low statistical power to exclude type II errors (Table 3), considering that the acceptable standard is ≥ 80%.

**Table 3:** Post-hoc analysis of statistical power.

**Author**	**Statistical power**
Calenda et al. [18]	21%
Calenda et al. [19]	24.9%
Calenda et al. [20]	4.1%

## Discussion

Until now, no systematic review has assessed bleeding risk during needle-based ophthalmic anesthesia in patients on antithrombotic therapy. Previous reviews generally advocated continuation of anticoagulants and antiplatelet agents during ophthalmic anesthesia, on the grounds that it conferred an increased risk only for minor bleeding [1, 7]. However, these reviews did not evaluate the methodology and risk of bias of the original studies, thereby limiting the validity of the conclusions [22, 23].

This systematic review investigated the available evidence regarding hemorrhagic complications in patients undergoing needle ophthalmic anesthesia while using antithrombotic agents. In the included studies, the incidence of mild to moderate hemorrhagic episodes related to needle-based ophthalmic block did not differ significantly between patients using vitamin K inhibitors, ASA, or clopidogrel and non-users of these medications. Furthermore, serious hemorrhagic complications were very rare, regardless of whether patients were on antithrombotic therapy or not. However, these findings should be attentively evaluated due to 1) the low statistical power shown in the post-hoc analysis to exclude type II errors in the experimental studies as well as 2) a large 95% CI of some subgroups of the two retrospective cohort studies. Post-hoc power analysis has been criticized while interpreting negative study results, and a 95% CI has been postulated as a better estimation tool for statistical power [24]. In this review, post-hoc power analysis and 95% CIs were evaluated to critically assess the results from the included studies. Caution should be taken regarding consideration of any differences in low and moderate hemorrhagic complications in the studied groups. However, the rare occurrence of severe hemorrhagic complications, while making it impossible to evaluate the statistical difference between patients using or not using antithrombotics, supports the safety profile of needle ophthalmic block in patients using antithrombotic agents, as described in the selected studies of this review.

Major perioperative cardiovascular complications, such as nonfatal myocardial infarction, nonfatal stroke, and death, occur in nearly 10 million patients annually [25]. Reduction of these rates involves a balance between risk of bleeding and risk of thromboembolism [3] when managing antithrombotic agents. In patients undergoing ophthalmic surgery, who are usually older and on chronic pharmacological therapy, this is especially important [2]. Nevertheless, ophthalmic surgery presents a low cardiac risk [26], although even in this setting, premature antiplatelet discontinuation after percutaneous coronary intervention for surgery is associated with an increase in cardiac events [27]. In a multicenter retrospective study conducted in 666 patients with coronary stents undergoing cardiac or non-cardiac surgery, perioperative antiplatelet discontinuation was the most important independent predictor of major cardiac complications (OR = 25.8) [4]. Moreover, ASA withdrawal precedes up to 10.2% of acute cardiovascular adverse events [8], perhaps as a result of a rebound effect resulting in higher platelet aggregation [9].

Chronic anticoagulation therapy is warranted in three major clinical situations: atrial fibrillation, valvular heart disease, and prophylaxis against venous thromboembolisms [1]. If a patient is at high risk of a thromboembolic event, perioperative anticoagulation should not be suspended, particularly if the surgery or invasive procedure is associated with a low risk of bleeding [3], as are most ophthalmic interventions [1]. For atrial fibrillation, suspension of anticoagulation for a short period before surgery does not seem to raise the risk of thromboembolic events [12].

Sub-Tenon’s block was not included in this review, as it is not classified in the same category of ophthalmic regional anesthesia as peribulbar and retrobulbar anesthesia [28]. Had it been included, the transconjunctival approach in sub-Tenon’s block would have acted as a confounding factor regarding the cause of hemorrhage. Similarly, with regard to hemorrhage related to ophthalmic regional anesthesia, subconjunctival and eyelid bleeding are classified separately [2, 5]. Eyelid hemorrhage was included in Kallio’s grade scale [16], developed to evaluate hemorrhagic complications related to peribulbar or retrobulbar block. On the other hand, studies assessing sub-Tenon’s anesthesia generally consider subconjunctival hemorrhage as a complication [2, 29]. This might be because sub-Tenon’s block demands a transconjunctival approach [28], raising the risk of subconjunctival hemorrhage [2]. In this regard, Benzimra et al. [2], retrospectively evaluating 48,862 cataract surgeries, found that subconjunctival hemorrhage was significantly increased in patients who underwent ophthalmic regional anesthesia, including sub-Tenon’s block, when they were using clopidogrel (4.39%) or warfarin (3.67%) compared with when they were not using any of these medications (1.68%). However, with regard to eyelid bleeding, no differences were found among groups, which is in agreement with the findings of the present review.

Severe hemorrhagic complication related to needle block ophthalmic anesthesia is a rare event [2]. Without considering the use of antithrombotics, Riad et al. [30] found a very low incidence of retrobulbar hemorrhages (0.01%) among 33,363 peribulbar blocks. In a study selected for this review, Katz et al. [21] reported a higher but still small incidence of retrobulbar hemorrhage (0.04%), although no difference was found between patients using and not using ASA. It is noteworthy that retrobulbar and peribulbar anesthesia were considered as a single regional block in this study. A Cochrane review [31] found no difference in efficacy and safety between the two techniques; however, this review was limited by its selection of only a few randomized studies, with only one on retrobulbar hemorrhage. With a large sample, peribulbar and retrobulbar anesthesia, studied separately, might present different profiles of complications. For instance, a higher incidence of retrobulbar hemorrhage might be found.

Few studies have evaluated dual antiplatelet therapy in patients undergoing ophthalmic surgery [1, 2]. No previous studies fulfilled the criteria for inclusion in this review. Benzimra et al. [2], with a sample of more than 48,000 patients, found 134 users of both clopidogrel and aspirin who underwent ophthalmic regional anesthesia (sub-Tenon’s block or retrobulbar/peribulbar block). The incidence of mild to moderate hemorrhagic complications in these patients was 3.74%, with no serious complications reported. Given the clinical relevance of antiplatelet agents after percutaneous coronary intervention [4], studies evaluating hemorrhagic complications related to ophthalmic regional anesthesia in patients on dual antiplatelet therapy would be advisable.

New oral anticoagulants (rivaroxaban, apixaban, and dabigatran) and antiplatelet agents (prasugrel and ticagrelor) were included in the search strategy of this review. However, no studies were found in the selected databases that evaluated bleeding risk in patients undergoing ophthalmic regional anesthesia while using these new antithrombotics. Future studies should address this gap in knowledge.

In summary, with significant limitations, the studies included in this review found no evidence of an increased risk of mild to moderate hemorrhagic complications related to needle-based ophthalmic anesthesia in patients using aspirin, clopidogrel, or vitamin K inhibitors as described in each individual study. The occurrence of severe hemorrhagic complication was very rare, regardless of whether patients were on antithrombotic therapy. Although statistically limited, these results suggest that needle ophthalmic anesthesia is not a cause for interruption of anticoagulants, antiplatelet agents, or other antithrombotics.

### Limitations

Low statistical power is a relevant issue limiting the results of all the included experimental studies to exclude type II error. In addition, the 95% confidence interval was large in some subgroups of the two retrospective cohorts, showing low strength of these studies.

High heterogeneity prevented meta-analysis of the results, which could increase the statistical power, but in the absence of synthesis, sample size was an important limitation. For an unusual event like retrobulbar hemorrhage (0.04%), a sample size of 20,000 patients would be necessary to determine whether ophthalmic anesthesia raises the incidence [21]. Further studies with enough power should be designed to overcome this issue.

Other limitation concerns included the technical aspects of needle-based ophthalmic regional anesthesia, including needle characteristics and the experience of the anesthesiologist, which can be very different among studies. These confounding features can be hard to evaluate, especially in a large multicenter retrospective cohort study. Finally, randomization is not feasible for these types of studies, as purposeful discontinuation of antithrombotics would unethically raise the risk of thrombotic events.

## Conclusions

In this review, none of the selected studies showed significant bleeding related to needle-based ophthalmic regional anesthesia in association with the use of aspirin, clopidogrel, or vitamin K inhibitors. However, it is not possible to make any statistically significant conclusions about whether antithrombotics increase the incidence of these events. Since the available data is not powerful enough to provide a reliable evaluation of the true effect of antithrombotics in this setting, new studies to address these limitations are necessary.

## Supporting Information

S1 PRISMA Checklist (DOCX)

S1 Table Database search strategy. (DOCX)

S2 Table Excluded articles with reasons for exclusion. (DOCX)

S3 Table Risk of bias assessed by Meta Analysis of Statistics Assessment and Review Instrument (MASTARI) critical appraisal tools. (DOCX)

## References

[1] Bonhomme F, Hafezi F, Boehlen F, Habre W. Management of antithrombotic therapies in patients scheduled for eye surgery. Eur J Anaesthesiol. 2013;30: 449–454. 10.1097/EJA.0b013e328360c442 23698703

[2] Benzimra JD, Johnston RL, Jaycock P, Galloway PH, Lambert G, Chung AK, et al The Cataract National Dataset electronic multicentre audit of 55,567 operations: antiplatelet and anticoagulant medications. Eye (Lond). 2009;23: 10–16. 18259210 10.1038/sj.eye.6703069

[3] Baron TH, Kamath PS, McBane RD. Management of antithrombotic therapy in patients undergoing invasive procedures. N Engl J Med. 2013;368: 2113–2124. 10.1056/NEJMra1206531 23718166

[4] Rossini R, Musumeci G, Capodanno D, Lettieri C, Limbruno U, Tarantini G, et al Perioperative management of oral antiplatelet therapy and clinical outcomes in coronary stent patients undergoing surgery. Results of a multicentre registry. Thromb Haemost. 2015;113: 272–282. 10.1160/TH14-05-0436 25274620

[5] Rizk SN, Fahim MR, El-Zakzouk ES. Peribulbar versus sub-Tenon block in cardiac patients undergoing cataract surgery during warfarin therapy. Egyptian Journal of Anaesthesia. 2014;30: 255–259.

[6] Kiire CA, Mukherjee R, Ruparelia N, Keeling D, Prendergast B, Norris JH. Managing antiplatelet and anticoagulant drugs in patients undergoing elective ophthalmic surgery. Br J Ophthalmol. 2014;98: 1320–1324. 10.1136/bjophthalmol-2014-304902 24692748

[7] Mather SJ, Kong KL, Vohra SB. Loco-regional anaesthesia for ocular surgery: Anticoagulant and antiplatelet drugs. Curr Anaesth Crit Care. 2010;21: 158–163.

[8] Burger W, Chemnitius JM, Kneissl GD, Rucker G. Low-dose aspirin for secondary cardiovascular prevention—cardiovascular risks after its perioperative withdrawal versus bleeding risks with its continuation—review and meta-analysis. J Intern Med. 2005;257: 399–414. 15836656 10.1111/j.1365-2796.2005.01477.x

[9] Smart S, Aragola S, Hutton P. Antiplatelet agents and anaesthesia. Continuing Education in Anaesthesia, Critical Care & Pain. 2007;7: 157–161.

[10] Levine GN, Bates ER, Blankenship JC, Bailey SR, Bittl JA, Cercek B, et al 2011 ACCF/AHA/SCAI Guideline for Percutaneous Coronary Intervention: executive summary: a report of the American College of Cardiology Foundation/American Heart Association Task Force on Practice Guidelines and the Society for Cardiovascular Angiography and Interventions. Catheter Cardiovasc Interv. 2012;79: 453–495. 10.1002/ccd.23438 22328235

[11] Kolh P, Windecker S, Alfonso F, Collet JP, Cremer J, Falk V, et al 2014 ESC/EACTS Guidelines on myocardial revascularization: the Task Force on Myocardial Revascularization of the European Society of Cardiology (ESC) and the European Association for Cardio-Thoracic Surgery (EACTS). Developed with the special contribution of the European Association of Percutaneous Cardiovascular Interventions (EAPCI). Eur J Cardiothorac Surg. 2014;46: 517–592. 10.1093/ejcts/ezu366 25173601

[12] Douketis JD, Spyropoulos AC, Kaatz S, Becker RC, Caprini JA, Dunn AS, et al Perioperative bridging anticoagulation in patients with atrial fibrillation. N Engl J Med. 2015;373: 823–833. 10.1056/NEJMoa1501035 26095867 PMC4931686

[13] Mason JO, Gupta SR, Compton CJ, Frederick PA, Neimkin MG, Hill ML, et al Comparison of hemorrhagic complications of warfarin and clopidogrel bisulfate in 25-gauge vitrectomy versus a control group. Ophthalmology. 2011;118: 543–547. 10.1016/j.ophtha.2010.07.005 20884061

[14] Kobayashi H. Evaluation of the need to discontinue antiplatelet and anticoagulant medications before cataract surgery. J Cataract Refract Surg. 2010;36: 1115–1119. 10.1016/j.jcrs.2010.01.017 20610088

[15] Moher D, Liberati A, Tetzlaff J, Altman DG. Preferred reporting items for systematic reviews and meta-analyses: the PRISMA statement. Int J Surg. 2010;8: 336–341. 10.1016/j.ijsu.2010.02.007 20171303

[16] Kallio H, Paloheimo M, Maunuksela EL. Haemorrhage and risk factors associated with retrobulbar/peribulbar block: a prospective study in 1383 patients. Br J Anaesth. 2000;85: 708–711. 11094585 10.1093/bja/85.5.708

[17] The Joanna Briggs Institute. Joanna Briggs Institute Reviewers’ Manual: 2014 edition Australia: The Joanna Briggs Institute; 2014.

[18] Calenda E, Cardon-Guiton A, Genevois O, Gueudry J, Muraine M. Peribulbar block in 500 patients scheduled for eye procedures and treated with acetyl salicylic acid. Acta Anaesthesiol Taiwan. 2011;49: 141–143. 10.1016/j.aat.2011.11.003 22221686

[19] Calenda E, Lamothe L, Genevois O, Cardon A, Muraine M. Peribulbar block in patients scheduled for eye procedures and treated with clopidogrel. J Anesth. 2012;26: 779–782. 10.1007/s00540-012-1406-6 22581096

[20] Calenda E, Genevois O, Cardon A, Muraine M. Peribulbar anesthesia in 750 patients treated with oral anticoagulants. Int J Ophthalmol. 2014;7: 110–113. 10.3980/j.issn.2222-3959.2014.01.20 24634874 PMC3949469

[21] Katz J, Feldman MA, Bass EB, Lubomski LH, Tielsch JM, Petty BG, et al Risks and benefits of anticoagulant and antiplatelet medication use before cataract surgery. Ophthalmology. 2003;110: 1784–1788. 13129878 10.1016/S0161-6420(03)00785-1

[22] Kong KL, Khan J. Ophthalmic patients on antithrombotic drugs: a review and guide to perioperative management. Br J Ophthalmol. 2015;99: 1025–1030. 10.1136/bjophthalmol-2014-306036 25425711

[23] Grzybowski A, Ascaso FJ, Kupidura-Majewski K, Packer M. Continuation of anticoagulant and antiplatelet therapy during phacoemulsification cataract surgery. Curr Opin Ophthalmol. 2015;26: 28–33. 10.1097/ICU.0000000000000117 25390860

[24] Levine M, Ensom MH. Post hoc power analysis: an idea whose time has passed? Pharmacotherapy. 2001;21: 405–409. 11310512 10.1592/phco.21.5.405.34503

[25] Darvish-Kazem S, Gandhi M, Marcucci M, Douketis JD. Perioperative management of antiplatelet therapy in patients with a coronary stent who need noncardiac surgery: a systematic review of clinical practice guidelines. Chest. 2013;144: 1848–1856. 10.1378/chest.13-0459 23928727

[26] Fleisher LA, Fleischmann KE, Auerbach AD, Barnason SA, Beckman JA, Bozkurt B, et al 2014 ACC/AHA guideline on perioperative cardiovascular evaluation and management of patients undergoing noncardiac surgery: executive summary: a report of the American College of Cardiology/American Heart Association Task Force on practice guidelines. Developed in collaboration with the American College of Surgeons, American Society of Anesthesiologists, American Society of Echocardiography, American Society of Nuclear Cardiology, Heart Rhythm Society, Society for Cardiovascular Angiography and Interventions, Society of Cardiovascular Anesthesiologists, and Society of Vascular Medicine Endorsed by the Society of Hospital Medicine. J Nucl Cardiol. 2015;22: 162–215. 10.1007/s12350-014-0025-z 25523415

[27] Schouten O, van Domburg RT, Bax JJ, de Jaegere PJ, Dunkelgrun M, Feringa HH, et al Noncardiac surgery after coronary stenting: early surgery and interruption of antiplatelet therapy are associated with an increase in major adverse cardiac events. J Am Coll Cardiol. 2007;49: 122–124. 17207733 10.1016/j.jacc.2006.10.004

[28] Nouvellon E, Cuvillon P, Ripart J. Regional anesthesia and eye surgery. Anesthesiology. 2010;113: 1236–1242. 10.1097/ALN.0b013e3181f7a78e 20938330

[29] Saumier N, Lorne E, Dermigny F, Walkzak K, Daelman F, Jezraoui P, et al [Safety of "needle" regional anaesthesia for anterior segment surgery under antiplatelet agents and anticoagulants therapies]. Ann Fr Anesth Reanim. 2010;29: 878–883. [Article in French] 10.1016/j.annfar.2010.08.009 21112731

[30] Riad W, Akbar F. Ophthalmic regional blockade complication rate: a single center audit of 33,363 ophthalmic operations. J Clin Anesth. 2012;24: 193–195. 10.1016/j.jclinane.2011.07.012 22459339

[31] Alhassan MB, Kyari F, Ejere HO. Peribulbar versus retrobulbar anaesthesia for cataract surgery. Cochrane Database Sys Rev. 2008;3:CD004083. 10.1002/14651858.CD004083.pub2 18646099

