# Supplementary material for: Risk of Hemorrhage during Needle-Based Ophthalmic Regional Anesthesia in Patients Taking Antithrombotics: A Systematic Review
Source: PLoS One. 2016 Jan 22;11(1):e0147227. doi: 10.1371/journal.pone.0147227 (PMC4723334; doi:10.1371/journal.pone.0147227)
Supplement: S1 Table — (DOCX) [file pone.0147227.s002.docx]

**S1 Table** Database search strategy.

| Databases | Search |
| --- | --- |
| Cochrane, LILACS, PubMed, Scopus, Web of Science, Google Scholar | (“OPHTALMIC ANESTHESIA” OR PERIBULBAR OR RETROBULBAR) AND (ANTICOAGULANTS OR ANTIPLATELET OR ACETYLSALICYLIC ACID OR CLOPIDOGREL OR TICLOPIDINE OR PRASUGREL OR TICAGRELOR OR WARFARIN OR DABIGATRAN OR APIXABAN OR RIVAROXABAN) AND (HEMORRHAGE) |
