# Supplementary material for: Risk of Hemorrhage during Needle-Based Ophthalmic Regional Anesthesia in Patients Taking Antithrombotics: A Systematic Review
Source: PLoS One. 2016 Jan 22;11(1):e0147227. doi: 10.1371/journal.pone.0147227 (PMC4723334; doi:10.1371/journal.pone.0147227)
Supplement: S2 Table — (DOCX) [file pone.0147227.s003.docx]

**S2 Table**. Excluded articles with reasons for exclusion.

| Author | Reason for exclusion |
| --- | --- |
| Benzimra ^1^ | 3 |
| Passemard ^2^ | 4 |
| Rizk^3^  Saumier ^4^ | 5  3 |

1- Studies in children 2- Reviews, letters, abstract conference, editorials 3- Subtenon’s block 4- Studies that hemorrhagic complication were associate to the surgery, and not the anesthesia 5- studies where it was not clearly stated if patients used antithrombotic agents or without a group control.

**REFERENCES**

1- Benzimra, J. D., R. L. Johnston, P. Jaycock, P. H. Galloway, G. Lambert, A. K. Chung, T. Eke, and J. M. Sparrow, 2009, The Cataract National Dataset electronic multicentre audit of 55,567 operations: antiplatelet and anticoagulant medications, Eye (Lond), v. 23: England, p. 10-6.

2- [Passemard M](http://www.ncbi.nlm.nih.gov/pubmed/?term=Passemard%20M%5BAuthor%5D&cauthor=true&cauthor_uid=22495328)1, [Koehrer P](http://www.ncbi.nlm.nih.gov/pubmed/?term=Koehrer%20P%5BAuthor%5D&cauthor=true&cauthor_uid=22495328), [Juniot A](http://www.ncbi.nlm.nih.gov/pubmed/?term=Juniot%20A%5BAuthor%5D&cauthor=true&cauthor_uid=22495328), [Bron AM](http://www.ncbi.nlm.nih.gov/pubmed/?term=Bron%20AM%5BAuthor%5D&cauthor=true&cauthor_uid=22495328), [Creuzot-Garcher C](http://www.ncbi.nlm.nih.gov/pubmed/?term=Creuzot-Garcher%20C%5BAuthor%5D&cauthor=true&cauthor_uid=22495328), 2012 Maintenance of anticoagulant and antiplatelet agents for patients undergoing peribulbar anesthesia and vitreoretinal surgery, Retina. 2012 Oct;32(9):1868-73

3- Rizk, Mona R. Fahim, Ehab S. El-Zakzouk. Peribulbar versus sub-Tenon block in cardiac patients undergoing cataract surgery during warfarin therapy. Egyptian Journal of Anaesthesia (2014) 30, 255–259

4- Saumier E. Lorne, F. Dermigny, K. Walkzak, F. Daelman, P. Jezraoui, Y. Mahjoub, S. Milazzo, H. Dupont, 2010 Changement de pratique en ALR ophtalmique (ALR a` l’aiguille et maintien des anticoagulants) : se ́curite ́ vis-a`-vis des incidents he ́morragiques mineurs, Annales Françaises d’Anesthesie et de Reanimation 29 (2010) 878–883
