# Supplementary material for: Risk of Hemorrhage during Needle-Based Ophthalmic Regional Anesthesia in Patients Taking Antithrombotics: A Systematic Review
Source: PLoS One. 2016 Jan 22;11(1):e0147227. doi: 10.1371/journal.pone.0147227 (PMC4723334; doi:10.1371/journal.pone.0147227)
Supplement: S3 Table — (DOCX) [file pone.0147227.s004.docx]

**S3 Table** Risk of bias assessed by Meta Analysis of Statistics Assessment and Review Instrument (MAStARI) critical appraisal tools. Risk of bias was categorized as High when the study reaches up to 49% score “yes”, Moderate when the study reached 50% to 69% score “yes”, and Low when the study reached more than 70% score “yes”.

3A - Descriptive

|  | Calenda et al.^1^ | Calenda et al.^2^ | Calenda et al.^3^ |
| --- | --- | --- | --- |
| 1. Was the study based on a random or pseudorandom sample? | N | N | N |
| 1. Were the criteria for inclusion in the sample clearly defined? | Y | Y | Y |
| 1. Were confounding factors identified and strategies to deal with them stated? | Y | Y | Y |
| 1. Were outcomes assessed using objective criteria? | Y | Y | Y |
| 1. If comparisons are being made, was there sufficient description of the groups? | Y | Y | Y |
| 1. Was the follow up carried out over a sufficient time period? | Y | Y | Y |
| 1. Were the outcomes of people who withdrew described and included in the analysis? | NA | NA | NA |
| 1. Were the outcomes measured in a reliable way? | Y | Y | Y |
| 1. Was an appropriate statistical analysis used? | Y | Y | Y |
| % yes/risk | 77.77/  low | 77.77/  low | 77.77/  low |

*Y=Yes, N=No, U=Unclear, NA=Not applicable

3B – Cohort Studies

|  | Kallio et al. ^4^ | Katz et al.^5^ |
| --- | --- | --- |
| 1. Is sample representative of patients in the population as a whole? | N | N |
| 1. Are the patients at a similar point in the course of their condition/illness | Y | Y |
| 1. Has bias been minimized in relation to selection of cases and of controls? | N | N |
| 1. Are confounding factors identified and strategies to deal with them stated? | N | N |
| 1. Are outcomes assessed using objective criteria? | Y | Y |
| 1. Was the follow up carried out over a sufficient time period? | Y | Y |
| 1. Were the outcomes of people who withdrew described and included in the analysis? | Y | Y |
| 1. Were the outcomes measured in a reliable way? | Y | Y |
| 1. Was an appropriate statistical analysis used? | Y | Y |
| % yes/risk | 66.66/  moderate | 66.66/  moderate |

**REFERENCES**

1- [Calenda E](http://www.ncbi.nlm.nih.gov/pubmed/?term=Calenda%20E%5BAuthor%5D&cauthor=true&cauthor_uid=22221686)1, [Cardon-Guiton A](http://www.ncbi.nlm.nih.gov/pubmed/?term=Cardon-Guiton%20A%5BAuthor%5D&cauthor=true&cauthor_uid=22221686), [Genevois O](http://www.ncbi.nlm.nih.gov/pubmed/?term=Genevois%20O%5BAuthor%5D&cauthor=true&cauthor_uid=22221686), [Gueudry J](http://www.ncbi.nlm.nih.gov/pubmed/?term=Gueudry%20J%5BAuthor%5D&cauthor=true&cauthor_uid=22221686), [Muraine M](http://www.ncbi.nlm.nih.gov/pubmed/?term=Muraine%20M%5BAuthor%5D&cauthor=true&cauthor_uid=22221686). Peribulbar block in 500 patients scheduled for eye procedures and treated with acetyl salicylic acid. Acta Anaesthesiol Taiwan. 2011 Dec;49(4):141-3. doi: 10.1016/j.aat.2011.11.003. Epub 2011 Dec 22.

2- [Calenda E](http://www.ncbi.nlm.nih.gov/pubmed/?term=Calenda%20E%5BAuthor%5D&cauthor=true&cauthor_uid=22581096)1, [Lamothe L](http://www.ncbi.nlm.nih.gov/pubmed/?term=Lamothe%20L%5BAuthor%5D&cauthor=true&cauthor_uid=22581096), [Genevois O](http://www.ncbi.nlm.nih.gov/pubmed/?term=Genevois%20O%5BAuthor%5D&cauthor=true&cauthor_uid=22581096), [Cardon A](http://www.ncbi.nlm.nih.gov/pubmed/?term=Cardon%20A%5BAuthor%5D&cauthor=true&cauthor_uid=22581096), [Muraine M](http://www.ncbi.nlm.nih.gov/pubmed/?term=Muraine%20M%5BAuthor%5D&cauthor=true&cauthor_uid=22581096). Peribulbar block in patients scheduled for eye procedures and treated with clopidogrel. J Anesth. 2012 Oct;26(5):779-82. doi: 10.1007/s00540-012-1406-6. Epub 2012 May 12

3- [Calenda E](http://www.ncbi.nlm.nih.gov/pubmed/?term=Calenda%20E%5BAuthor%5D&cauthor=true&cauthor_uid=24634874)1, [Genevois O](http://www.ncbi.nlm.nih.gov/pubmed/?term=Genevois%20O%5BAuthor%5D&cauthor=true&cauthor_uid=24634874)2, [Cardon A](http://www.ncbi.nlm.nih.gov/pubmed/?term=Cardon%20A%5BAuthor%5D&cauthor=true&cauthor_uid=24634874)1, [Muraine M](http://www.ncbi.nlm.nih.gov/pubmed/?term=Muraine%20M%5BAuthor%5D&cauthor=true&cauthor_uid=24634874)2. Peribulbar anesthesia in 750 patients treated with oral anticoagulants. Int J Ophthalmol. 2014 Feb 18;7(1):110-3. doi: 10.3980/j.issn.2222-3959.2014.01.20. eCollection 2014.

4- [Kallio H](http://www.ncbi.nlm.nih.gov/pubmed/?term=Kallio%20H%5BAuthor%5D&cauthor=true&cauthor_uid=11094585)1, [Paloheimo M](http://www.ncbi.nlm.nih.gov/pubmed/?term=Paloheimo%20M%5BAuthor%5D&cauthor=true&cauthor_uid=11094585), [Maunuksela EL](http://www.ncbi.nlm.nih.gov/pubmed/?term=Maunuksela%20EL%5BAuthor%5D&cauthor=true&cauthor_uid=11094585). Haemorrhage and risk factors associated with retrobulbar/peribulbar block: a prospective study in 1383 patients. Br J Anaesth. 2000 Nov;85(5):708-11.

5- [Katz J](http://www.ncbi.nlm.nih.gov/pubmed/?term=Katz%20J%5BAuthor%5D&cauthor=true&cauthor_uid=13129878)1, [Feldman MA](http://www.ncbi.nlm.nih.gov/pubmed/?term=Feldman%20MA%5BAuthor%5D&cauthor=true&cauthor_uid=13129878), [Bass EB](http://www.ncbi.nlm.nih.gov/pubmed/?term=Bass%20EB%5BAuthor%5D&cauthor=true&cauthor_uid=13129878), [Lubomski LH](http://www.ncbi.nlm.nih.gov/pubmed/?term=Lubomski%20LH%5BAuthor%5D&cauthor=true&cauthor_uid=13129878), [Tielsch JM](http://www.ncbi.nlm.nih.gov/pubmed/?term=Tielsch%20JM%5BAuthor%5D&cauthor=true&cauthor_uid=13129878), [Petty BG](http://www.ncbi.nlm.nih.gov/pubmed/?term=Petty%20BG%5BAuthor%5D&cauthor=true&cauthor_uid=13129878), [Fleisher LA](http://www.ncbi.nlm.nih.gov/pubmed/?term=Fleisher%20LA%5BAuthor%5D&cauthor=true&cauthor_uid=13129878), [Schein OD](http://www.ncbi.nlm.nih.gov/pubmed/?term=Schein%20OD%5BAuthor%5D&cauthor=true&cauthor_uid=13129878); [Study of Medical Testing for Cataract Surgery Team](http://www.ncbi.nlm.nih.gov/pubmed/?term=Study%20of%20Medical%20Testing%20for%20Cataract%20Surgery%20Team%5BCorporate%20Author%5D). Risks and benefits of anticoagulant and antiplatelet medication use before cataract surgery. Ophthalmology. 2003 Sep;110(9):1784-8.
